# Repeatability and Variability of a High-Fat High-Fructose Diet-Induced Metabolic Syndrome Model in Young Adult Male Wistar Rats

**DOI:** 10.3390/mps9010007

**Published:** 2026-01-06

**Authors:** Danail Pavlov, Silvia Gancheva, Klementina Moneva-Marinova, Antoaneta Georgieva, Milena Todorova, Nadezhda Stefanova, Mehmed Reyzov, Elis Rafailova, Miroslav Eftimov, Maria Tzaneva, Stefka Valcheva-Kuzmanova, Maria Zhelyazkova-Savova

**Affiliations:** 1 Department of Biochemistry, Medical University of Varna, 55 Marin Drinov Str., 9002 Varna, Bulgaria; 2 Department of Pharmacology and Clinical Pharmacology and Therapy, Medical University of Varna, 55 Marin Drinov Str., 9002 Varna, Bulgaria; silvia.gancheva@mu-varna.bg (S.G.); klementina.moneva@mu-varna.bg (K.M.-M.); antoaneta.georgieva@mu-varna.bg (A.G.); milena.todorova@mu-varna.bg (M.T.); mehmed.abtulov@mu-varna.bg (M.R.); elis.rafailova@mu-varna.bg (E.R.); miroslav.eftimov@mu-varna.bg (M.E.); stefkavk@yahoo.com (S.V.-K.); mariadz52@yahoo.com (M.Z.-S.); 3 Department of General and Clinical Pathology, Medical University of Varna, 55 Marin Drinov Str., 9002 Varna, Bulgaria; nadejda.stefanova@mu-varna.bg (N.S.); mtzaneva@hotmail.com (M.T.)

**Keywords:** in vivo pharmacological models, metabolic syndrome, Wistar rats, high-fat high-fructose diet

## Abstract

Metabolic syndrome is a disorder of energy metabolism characterized by persistently high prevalence and significant medical and economic burden on society. An effective animal model that closely replicates the key features of the syndrome in humans is essential for evaluating therapeutic strategies aimed at improving health outcomes. High-calorie diet-induced animal models of metabolic syndrome are preferred by many research groups for studying its pathogenesis, prevention and therapy. However, there are numerous variations in the types and proportions of carbohydrates and/or fats in the diets used. In 2015, our research team developed a diet-induced model of metabolic syndrome in young adult male Wistar rats that was based on adding 17% animal fat and 17% fructose to the standard rat chow and 10% fructose to the drinking water. This model reliably induced the morphometric and biochemical alterations that represent the core diagnostic features of the syndrome in humans. Since its initial introduction, we have utilized the high-fat high-fructose diet-induced model of metabolic syndrome/obesity in ten experimental studies. The current paper provides a protocol for applying the model, presents its repeatability and discusses the variability in the morphometric, biochemical, histopathological, immunohistochemical, and behavioral data of 10 experimental studies on Wistar rats.

## 1. Introduction

Metabolic syndrome is a disorder of energy utilization and storage characterized by visceral obesity, dyslipidemia, arterial hypertension, and impaired glucose tolerance with insulin resistance, according to the criteria defined by the World Health Organization (WHO) and the International Diabetes Federation (IDF) [1]. Its global prevalence remains consistently high, ranging from 12.5% to 31.4%, depending on the diagnostic criteria. Central obesity, the most significant factor associated with the development of the syndrome, has a prevalence of 45.1% [2]. The medical, social, and economic impact of metabolic syndrome and obesity is considerable, not only because of their widespread occurrence but also due to their well-established role as risk factors for major non-communicable diseases, including type 2 diabetes [3,4], Metabolic Dysfunction-Associated Steatotic Liver Disease (MASLD) [5,6], cardiovascular [7] and neuropsychiatric disorders [8,9,10,11,12], and certain malignancies [13].

Among the complications associated with metabolic syndrome, MASLD is one of the most prevalent. The link between metabolic syndrome and MASLD is particularly strong, with studies showing that approximately 88% of patients with non-alcoholic steatohepatitis exhibit clinical features of the syndrome [5]. Insulin resistance is considered the central mechanism connecting disrupted energy metabolism with structural and functional liver damage [6].

Metabolic syndrome is associated with significant cardiovascular risk. Each of the components of metabolic syndrome is a separate risk factor and the combination of them further accelerates atherosclerosis and elevates the rate and severity of CV disease.

The association between metabolic syndrome and neuropsychiatric disorders has been recognized more recently, but it also appears to be notably strong. Meta-analyses have shown that metabolic syndrome is more prevalent among individuals with depression compared to non-depressed populations [8] and that the relationship between depression and obesity is bidirectional [9]. Similarly, studies have demonstrated a significant association between metabolic syndrome and anxiety disorders [10,11]. In contrast, the link between metabolic syndrome and cognitive impairment in humans is less clearly defined. However, a meta-analysis concluded that being obese during midlife also significantly increases the risk of developing dementia later in life [12].

Experimental models of metabolic syndrome are valuable tools for studying the pathogenesis, prevention, and treatment of the condition. An effective experimental model should replicate the pathogenesis and key clinical features of the syndrome as it occurs in humans. Ideally, it should replicate the primary morphometric and biochemical alterations seen in human metabolic syndrome, including visceral obesity, disturbances in carbohydrate metabolism, and dyslipidemia. A further advantage of an animal model would be the manifestation of organ-specific damage commonly associated with the syndrome in humans. This may include histopathological liver alterations resembling MASLD; endothelial dysfunction reflecting cardiovascular compromise; and behavioral changes indicative of anxiety, depression, or cognitive impairment. Validating such an animal model would establish a critical platform for evaluating therapeutic strategies, including dietary interventions and pharmacological treatments, with the goal of enhancing human health outcomes.

Various models of metabolic syndrome have been described in the literature [14,15,16], including diet-induced [17], chemically induced [18,19], and genetically modified animal models [20]. Genetically modified models tend to offer high reproducibility and low variability in measured outcomes. However, the etiology and pathogenesis of the induced morphometric and biochemical changes in these models differ significantly from those observed in most human cases of metabolic syndrome.

In humans, the primary cause of metabolic syndrome appears to be the sedentary lifestyle [21] and unhealthy diet characterized by excessive caloric intake, commonly referred to as the “Western” diet [22]. Diet-induced models successfully mimic the human etiology and pathogenesis of the syndrome and are therefore favored by many research groups [17]. These models typically involve feeding animals a high-calorie diet rich in simple carbohydrates [23,24,25,26,27,28], animal fats [29,30,31,32], or both [33,34,35]. However, there is considerable variation in the types and proportions of carbohydrates and fats used in these diets [14,15,16,17]. Less frequently, models relying on dietary choline and methionine deficiency are utilized. They successfully induce the features of MASLD, but usually fail to reproduce core features of the syndrome, such as visceral obesity and insulin resistance [36,37].

Fructose is often the preferred carbohydrate source due to its well-known lipogenic effects, which include elevating serum triglyceride levels, promoting hepatic triglyceride accumulation, and increasing visceral fat deposits [38,39]. Unlike glucose, fructose does not stimulate insulin secretion, likely due to the absence of the fructose transporter GLUT5 in the pancreas [39,40,41]. Since insulin plays a key role in regulating food intake and body weight, high fructose consumption is associated with increased energy intake and weight gain [39]. High-fructose diets vary in composition, with fructose content ranging from 10% [23,24] to 60% [26], either added to the food or drinking water. The duration of dietary interventions also varies widely, from 3 [23,25] to 16 weeks [42]. Some researchers use sucrose instead of fructose [27,28]. Sucrose is a disaccharide of fructose and glucose, which are absorbed separately after enzymatic cleavage by sucrase in the intestine. The fructose is stated as the primary component responsible for the development of metabolic syndrome in sucrose-fed animals [15].

Fat-enriched diets are less frequently used but remain relevant. Depending on the fat contribution to total energy intake, such diets are categorized as high-fat (30–50%) or very high-fat (over 50%) [43]. High-fat diets are primarily employed to model visceral obesity, although they can also induce other features of metabolic syndrome. The severity of obesity increases in a dose-dependent manner with the fat content in the diet [31]. The type of fat used is also crucial; animal-derived fats such as lard and beef tallow are generally more effective in inducing metabolic syndrome features than plant-derived fats [16,29]. A possible approach is the additional enrichment of the high-fat diet with cholesterol and/or cholic acid. The fat induces visceral obesity and insulin resistance; cholesterol augments the development of dyslipidemia, atherosclerosis, and liver damage; and cholic acid significantly improves fat and cholesterol absorption, while additionally exacerbating atherosclerosis and the features of MASLD [44,45,46].

Another dietary approach involves combining high-fat and high-fructose elements [33,34,35]. High-fat high-fructose (HFHF) diets combine the hypertriglyceridemic effect of fructose and the obesity-inducing properties of dietary fat. An additional advantage of HFHF diets is that they mimic more closely the unhealthy “Western” human diet [14,15,16]. However, commercially available hypercaloric diets used to model obesity and metabolic syndrome in animals typically contain a very high-fat content, providing between 45% and 60% of total energy intake. This significantly exceeds the fat proportion in unhealthy human diets, which is generally around 35% [47]. The primary limitation of diet-induced models is their low reproducibility and high variability of the measured outcomes. The various components of metabolic syndrome—such as visceral obesity, insulin resistance with hyperglycemia and/or impaired glucose tolerance, and dyslipidemia—are reproduced to different extents by different research groups [15,17].

In addition to the dietary approach, the genetic susceptibility of the experimental animals to develop the features of metabolic syndrome is also essential for the success of the model applied. In the case of rats, Wistar Ottawa Karlsburg W and WNIN strains are more prone to obesity, insulin resistance, and dyslipidemia. However, the Sprague–Dawley and Wistar rats are the most frequently used strains for studying obesity because they are readily available. The main disadvantage of these strains is that the weight gain response and visceral fat accumulation are variable. Some animals gain weight rapidly, while others gain the same amount as rats fed a standard or low-fat diet, which provides a rationale for the incorporation of terms such as obesity-susceptible and obesity-resistant populations [48]. This diversity could be accepted as an additional advantage of these rat strains, since it might represent what happens in the development of human obesity [49]. The initial age of the experimental animals is an additional factor that influences the measured outcomes. For example, post-weaning rats develop fat mass accumulation and hyperglycemia more rapidly and effectively compared to young adult rats and their use is less time-consuming in experimental settings [50]. On the other hand, obesity and metabolic syndrome are not so typical at such a young age in humans, and since the purpose of experimental research is usually translational research, it is more justified to use adult rats.

In 2015, our research team developed an HFHF diet-induced model of metabolic syndrome in young adult male Wistar rats. The dietary regimen included standard chow enriched with 17% animal fat and 17% fructose, as well as 10% fructose dissolved in the drinking water [51]. These moderate proportions of fat and fructose were chosen to reflect the levels of animal fats and simple carbohydrates typically found in an unhealthy human diet. Over the past decade, we have applied this model in ten independent experimental studies to investigate various problems of interest, related to metabolic syndrome, such as the effects of the diet on animal behavior and cognitive functions [52,53], the impact of vitamin K [53,54,55], and the pathophysiological role of the vitamin K-dependent protein osteocalcin in disorders of energy metabolism [53,56,57,58,59], as well as the effects of plant products and biologically active substances of plant origin on different components of metabolic syndrome [60,61,62,63,64,65,66,67,68,69,70,71,72,73,74,75,76,77].

The current paper presents a detailed protocol for implementing the model, highlights its repeatability, and examines the variability in morphometric, biochemical, histopathological, immunohistochemical, and behavioral findings observed across these studies.

## 2. Materials and Methods

### 2.1. Experimental Animals

The experimental studies were conducted on young adult male Wistar rats aged between 8 and 10 weeks, with an average initial body weight of 220–280 g, 10 or 12 animals per group. The rats were housed in plastic cages, 5 or 6 animals per cage, under standard laboratory conditions: a 12 h light/dark cycle and an average room temperature of 22 ± 2 °C. The animals had free access to food and drinking water or fructose solution, except during fasting periods required for the glucose tolerance test (GTT) or insulin tolerance test (ITT). The duration of the experiments ranged from 8 to 15 weeks, with most studies lasting 10 weeks. All procedures involving animals were carried out under national and international regulations, including the EU Directive 2010/63/EU on the protection of animals used for scientific purposes. The animal study protocols were approved by the Ethics Committee of the Bulgarian Food Safety Agency (protocols 19/2011, 140/2016, 177/2017 and 204/2017) for studies involving animals. The experimental design of the studies is summarized in Table 1.

### 2.2. Preparation of the Diet

The HFHF diet used in our studies was prepared by supplementing standard rat chow (produced by a Bulgarian forage manufacturer and used at the Vivarium of the Medical University of Varna) with lard and fructose. According to the manufacturer’s formulation, the standard chow contained 20.48 g of protein (29% kcal), 3 g of fat (10% kcal), and carbohydrates (61% kcal) in the form of starch (38.3 g) and sugars (4.32 g) per 100 g, providing a total energy value of 279 kcal per 100 g. The content of vitamins and minerals was as follows: calcium—0.82%, phosphorus—0.66%, sodium—0.16%; iron—19.5 mg, copper—2.5 mg, iodine—0.153 mg, manganese—14.6 mg, zinc—12 mg, selenium—0.046 mg; vitamin A—1000 IU, vitamin D_3_—300 IU, vitamin C—3 mg per 100 g.

To prepare the HFHF diet, enriched with 17% animal fat and 17% fructose, 100 g of lard (of subcutaneous origin) and 100 g of fructose were placed in a water bath at a water temperature of 90 °C. After melting the lard and dissolving the fructose, they were mixed with 400 g of standard chow, ensuring their even distribution. The resulting diet was cooled to room temperature and stored in 200 g portions at 0–4 °C for no longer than 3 days. The HFHF diet provides 405 kcal per 100 g, with 38% of the energy derived from added fat and 4% from the fat in the standard chow, 20% from added fructose and 24% from the carbohydrates in the standard chow, and 14% from proteins. The fat and sugar contents were increased from 3 g and 4.32 g, respectively, in the standard diet to 18.7 g and 19.55 g per 100 g in the HFHF diet.

In addition to the HFHF diet, the experimental animals received a 10% fructose solution instead of plain drinking water. This solution was prepared ex tempore by dissolving 100 g of fructose in up to 1000 mL of tap water. Each 100 mL of the fructose solution provided an additional 40 kcal.

### 2.3. Conduct of the Studies

Animals were allocated to experimental groups in such a way as to ensure similar average initial body weight. Food (g) and water or fructose solution (mL) consumption per cage was recorded daily and averaged per animal. Caloric intake (CI) for the control animals was calculated using the formula:

CI [kcal] = 2.79 × food consumption [g]

For the HFHF group, caloric intake (CI) was calculated as:

CI [kcal] = 4.05 × food consumption [g] + 0.4 × fructose solution consumption [mL]

Body weight was recorded once, twice, or three times per week, depending on the specific experimental design.

In all experiments conducted, biochemical analyses, behavioral tests, histological evaluations, and immunohistochemical examinations were carried out following the same protocols, as detailed in the sections below.

Biochemical tests evaluating carbohydrate metabolism (glucose tolerance test, GTT and/or insulin tolerance test, ITT) were performed after at least 7 weeks on the diet. ITT was performed after 4 or 6 h of fasting. The animals were injected intraperitoneally with regular insulin at a dose of 0.75 UI/kg and blood glucose level was measured by a glucometer immediately before the injection and at the 30th, 60th and 90th minute. The GTT was conducted following overnight fasting. The animals were injected intraperitoneally with 40% glucose at a dose of 2 g/kg and blood glucose level was measured by a glucometer immediately before the injection of glucose and at the 30th, 60th and 90th minute. The blood glucose level measured immediately before insulin or glucose administration was defined as the fasting blood glucose level.

Behavioral and cognitive function tests were performed during the final week of the experiments, at least 2 days after GTT or ITT. The first behavioral test performed was always the open field test (OFT), used to assess locomotor activity and anxiety-like behavior [78,79]. The animals were placed in a novel arena, and the total distance moved, the number of times they stood on their hind limbs and time spent in the central vs. peripheral zones were recorded for 5 min as indicators of exploration and anxiety levels. The social interaction test (SIT) was also used to evaluate anxiety-like behavior [80]. In this test, the subject was placed in an arena with an unfamiliar partner, and the time spent in active social interaction (e.g., sniffing, following) was measured, with reduced interaction considered a marker of social withdrawal or increased anxiety. The novel object location test (NOLT) evaluated spatial memory [81]. Following the familiarization phase with two identical objects, one object was moved to a new location during the test phase, and increased exploration of the relocated object reflected intact spatial memory. The final behavioral test performed was the forced swim test (FST), used to detect depressive-like behavior [82]. Animals were placed in an inescapable container filled with water and thus forced to swim for 5 min. The duration of immobility was measured (no additional activity other than that required to keep the rat’s head above the water), with prolonged immobility interpreted as behavioral despair.

At the end of the experiments, under ether anesthesia, blood samples were collected from the sublingual veins for biochemical analyses. Animals were then euthanized by cervical dislocation. The serum was prepared by centrifugation of the blood at 2000 rpm for 10 min and stored at −20 °C until analysis. Retroperitoneal fat, and in some studies, mesenteric, paranephric, and perigonadal fat pads were dissected and weighed.

Fat indices were calculated using the following formula and reported in per mille (‰) to provide a convenient numeric scale:

Fat index = (fat pad weight [g]/body weight [g]) × 1000

Serum levels of triglycerides, total cholesterol, and HDL-cholesterol, as well as the hepatic enzymes alanine aminotransferase (ALT), aspartate aminotransferase (AST), and alkaline phosphatase (ALP), were measured using colorimetric kits with an AURIS 2021 spectrophotometer (Cecil Instruments Ltd., Cambridge, United Kingdom). Hepatic triglycerides were determined in 10% tissue homogenates prepared in the TRIS buffer. Levels of insulin, leptin, undercarboxylated osteocalcin, and the activity of the enzyme superoxide dismutase were measured in selected experiments using ELISA kits.

For histopathological evaluation, samples from the liver, retroperitoneal adipose tissue, heart, and coronary arteries were collected, fixed in 10% neutral buffered formalin, and embedded in paraffin (melting point: 52–54 °C) to prepare paraffin blocks. Sections 5 μm thick were cut and stained with hematoxylin and eosin (H&E) using standard histological protocols to assess morphological alterations [60,61,62,65,66,67].

Immunohistochemical analysis of liver and retroperitoneal adipose tissue samples was conducted using an indirect immunoperoxidase method with the mini KIT high pH (DAKO K8024). Expression of the apoptotic markers Bax and Bcl-2 was evaluated using rabbit polyclonal antibodies (1:50 dilution; Santa Cruz Biotechnology, Dallas, TX, USA), while the expression of the inflammatory marker MAC387 was assessed using a mouse monoclonal antibody (1:50 dilution; Santa Cruz Biotechnology, Dallas, TX, USA). Negative controls included sections incubated with normal serum. Detection was performed using the EnVision FLEX system. Images were acquired with the Leica Aperio ScanScope AT2 (Leica Biosystems, Nussloch, Germany) and analyzed using ImageScope v12.1.0.5029 software (Leica Biosystems, Nussloch, Germany). Semi-quantitative analysis of Bax, Bcl-2, and MAC387 expression was performed in 50 cells per sample, using a two-grade expression scoring system (0–1) for the adipose tissue [60,62,65] and a four-grade expression scoring system (1–4) for the liver [60,61,67], with overall immunoreactivity calculated as a weighted average. The Bax/Bcl-2 ratio was computed to estimate the relative apoptotic tendency of cells.

### 2.4. Statistical Analysis

For the statistical analysis of the results from the various experiments, different statistical methods were employed, including Student’s *t*-test for comparing a single parameter between two groups; one-way ANOVA followed by post hoc tests, such as Dunnett’s and Bonferroni’s post tests for comparing a single parameter among more than two groups; and two-way ANOVA for evaluating the effects of two independent variables across multiple groups. Statistical analyses were performed using GraphPad Prism software, version 5.0 (GraphPad Software, Inc., San Diego, CA, USA). A *p*-value < 0.05 was considered statistically significant. The numerical data presented in the current article illustrate the percentage change in the respective parameter measured in the groups subjected to the HFHF diet, relative to the control groups in each of the conducted experiments. The calculations are performed using the formula:

% change = (value in HFHF group/value in control group) × 100 − 100

Having calculated the % of change of each measured parameter in each experiment, the average % of change in all the experiments was calculated. As the number of animals in each experiment was not the same (*n* = 10 or 12), the weighted average for each parameter was determined as follows: each percentage was multiplied by its sample size, the sum was calculated and divided by the sum of all sample sizes.

## 3. Results and Discussion

### 3.1. Morphometric Outcomes

The final body weight of Wistar rats subjected to the HFHF diet was compared to the final body weight of the control animals in all ten experiments (Table 2). In all experiments, the final body weight of animals was not increased after consuming the HFHF diet.

According to some authors, a hypercaloric diet is more likely to produce body weight gain (BWG) than an isocaloric diet [83]. Even though the applied HFHF diet in our studies was hypercaloric, the relatively short duration of the experiments (between 8 and 15 weeks) was most probably the key factor contributing to the lack of significant BWG. In contrast, the retroperitoneal fat pads increased in 80% of experiments, with an average increase of almost 60%, indicating the development of visceral obesity (Table 3).

The results from the application of the HFHF diet in our studies were highly repeatable, linked to unchanged normal body weight, and increased visceral adiposity phenotype in rats. These findings are consistent with data from the available literature. In a comparable study, male Wistar rats fed with a high-fat, high-sugar diet for 20 weeks did not exhibit an increase in body weight compared to the control group until week 16 [84]. The same study showed an increase in retroperitoneal fat deposits, which is consistent with our results. A study using an HFHF diet similar to ours, but with a higher fructose concentration in the drinking water, conducted on male Wistar rats of comparable age and initial body weight to our experimental animals, reported no difference in the final body weight between the control and the experimental group; an increase in visceral adipose tissue, including mesenteric, epididymal, and retroperitoneal fat was, however, observed [85]. Similar findings regarding the absence of increased BWG in animals subjected to hypercaloric diets have also been reported by several other research groups. In these studies, despite the lack of significant changes in body weight, the animals developed biochemical abnormalities characteristic of metabolic syndrome, such as hypertriglyceridemia, impaired glucose tolerance, hyperinsulinemia, and insulin resistance [17,23,86]. Based on the aforementioned findings, our HFHF diet could be considered a proper approach to model the common clinical scenario, where visceral adiposity is observed without an increase in body mass index, typically seen in patients with metabolic syndrome.

A study found that metabolic syndrome risk factors were present with high prevalence in individuals with normal body weight. Surprisingly, metabolic syndrome was diagnosed in more than 17% of them [87].

It should be noted that normal-weight obesity significantly increases the risk of cardiovascular diseases and type 2 diabetes [88]. In our experimental model, both the pathogenesis and the achieved phenotype closely resemble human disease. We also presume that achieving this combination of features is related to the specific content of the HFHF diet applied. While numerous reports are linking high carbohydrate intake with metabolic syndrome, the type of carbohydrates and their application seem to be of primary significance. For example, clinical evidence suggests that high-carbohydrate high-fiber intake contributes to the amelioration of major features of metabolic syndrome [15]. When the beneficial effect of fibers is not present, though, chronic high carbohydrate consumption tends to be associated with the development of obesity, diabetes, hypertension and cardiovascular disease, among others [89]. While different carbohydrates such as fructose, glucose or sucrose could be used, the superiority of fructose as a means to induce metabolic syndrome in experimental animals has been manifested unequivocally [15]. Fructose has been continuously shown to adversely affect metabolic health regardless of weight gain [89]. This is largely explained by the specifics of its metabolism, conferring lipogenic properties to this monosaccharide. Apart from the carbohydrate type, the choice of its concentration is also of great importance. The concentration of fructose used in our experiments is found on the lower side of the range usually used in experimental models of metabolic syndrome in order to closely mimic the actual percentages of the caloric intake of humans that is represented by added sugars [90]. This can be one explanation for the lack of BWG in our experiments. While some studies report no impact of the type of fat included in the diet on body weight and composition [91], many authors observe that animal fats more effectively induce the features of metabolic syndrome as compared to plant-derived fats [29], thus the choice of lard as the main lipid component in our diet.

According to some authors, high-fructose diets may fail to increase BWG due to metabolic compensation, manifested by elevated energy expenditure in response to increased caloric intake [92]. Another possible explanation for the absence of significant changes in final body weight, despite the development of visceral obesity, is an alteration in body composition—specifically, a reduction in lean mass as a consequence of high-fat dietary manipulation [93]. This reduction in muscle mass may result from the lower protein content of the HFHF diet used, as increasing the proportion of carbohydrates and fats inevitably leads to a relative decrease in dietary protein.

An additional point of interest in our findings is the attenuated visceral fat accumulation observed in Experiments 9 and 10, particularly in the 12-week experiment 9. In this experimental series, the retroperitoneal fat index increased by only 25.71%, and the total visceral fat index by 13.44% (Table 3), values considerably lower than those observed in most other experiments. This highlights a degree of inter-experimental variability inherent to diet-induced metabolic syndrome models, even when standardized nutritional and housing conditions are maintained. Such variability may arise from multiple interacting factors, including differences in initial body weight (which can influence subsequent adiposity and energy balance [94], small variations in food consumption (even with apparently identical diet composition) [95], and environmental micro-variations (e.g., cage location, noise, ambient temperature gradients)—for instance, housing temperature has been shown to affect metabolic rate, thermogenesis, and fat accumulation in rodents [96]— all of which can significantly modulate metabolic outcomes in laboratory animals.

In addition to these variables, potential seasonal influences warrant consideration. Although not directly investigated here, laboratory rats retain circannual and photoperiodic sensitivity that can influence food intake and adipose tissue dynamics [97]. Experiments 9 and 10 were performed during the winter–early spring transition, a period associated in some rodent studies [97] with reduced fat accretion and altered energy balance. While this explanation remains speculative, acknowledging potential seasonal effects provides a broader context for understanding the variability observed in our model. Future work incorporating explicit photoperiodic controls or balanced seasonal sampling may help clarify the extent to which seasonal cues influence the repeatability and magnitude of metabolic syndrome development.

In all 7 experiments where food consumption was measured, this indicator decreased by more than 30% (Table 2). These results can be considered attributable to the higher content of saturated fats in the used HFHF diet [98]. We also observed increased consumption of liquids in all experiments, with an average rise of 45%. This increase is most probably related to the high palatability of the fructose solution or its addictive properties [99]. As evident from Table 2, the caloric intake in all the 7 experiments, where it was measured, was higher in the HFHF diet-exposed group than in the control group, with an average increase of 23%. The increased caloric intake could be due to fructose’s inability to induce satiety adequately corresponding to the amount of calories ingested because of the metabolic properties of fructose [90].

### 3.2. Biochemical Outcomes

Plasma triglyceride (TG) levels were measured in nine experiments and increased levels were found in seven of them (77.8%) with an average increase of approximately 60% (Table 4). Increased levels of plasma total cholesterol and fasting blood glucose were observed in three (37.5%) and four (50%) out of eight experiments, respectively. A GTT was performed in seven experiments and impaired glucose tolerance was found in five of them (71.4%). In two out of three ITT performed, plasma glucose level was elevated. Increased plasma triglyceride level, as well as elevated glucose levels during GTT and ITT, appear to be repeatable biochemical markers throughout our experiments. Both the increase of fasting blood glucose and total cholesterol levels are less repeatable. However, hypercholesterolemia is not included in the criteria for metabolic syndrome diagnosis.

Other biochemical markers were measured during some of the experiments according to the specific aims. These changes included increased LDL- and decreased HDL-cholesterol levels [53,54,58], increased atherogenic indices (TG/HDL-cholesterol and total cholesterol/HDL-cholesterol) [51,53,58], increased levels of liver TG [51,53], increased insulin [53,54,56], leptin [53,54], and serum and brain thiobarbituric acid-reacting substances (TBARS) levels [52,53], changes in superoxide dismutase activity [60,64,67,70], decreased undercarboxylated osteocalcin [53,56,59], as well as increased levels of the liver enzymes ALT, AST, ALP [67,77].

The comparison of the metabolic consequences of different high-calorie diets shows that the fat-enriched diets lead to significant weight gain, visceral fat accumulation, hypertriglyceridemia, hyperglycemia, hyperinsulinemia and insulin resistance, as reported by an overview on diet-induced metabolic syndrome models [83]. Fat- and carbohydrate-enriched diets are almost similar, with somewhat less effect on fasting blood glucose and insulin levels. Carbohydrate-rich diets do not dramatically change weight gain and fasting blood glucose levels, and significantly increase other biochemical markers [83]. These findings correspond to a large extent to our results: increased TG levels were observed in 81% of the articles regarding high-carbohydrate high-fat diets [83], which is extremely close to our result of 77.8%. According to some authors [100], there is a strong association between fat-enriched diets and oxidative stress. A positive correlation and even a bidirectional link between oxidative stress and metabolic syndrome has been found [101]. These correlations correspond to our experimental data. Increased levels of biochemical markers for liver damage (ALT, AST, ALP) were found in two of our experiments [67,77]. The HFHF diet has been shown to most closely resemble the parameters of human MASLD and is considered as a reliable model of diet-associated liver damage [102]. Increased leptin levels were found in Wistar rats subjected to carbohydrate- and fat-enriched diets, but not in those subjected to a high-fat diet [103]. The increased leptin levels in two of our experiments [53,54] could be related to the high fructose intake, as studies have reported such an association [104].

According to the general definitions of metabolic syndrome in humans, as outlined by the IDF and WHO, its core features include visceral (central) obesity, insulin resistance, characteristic alterations in the lipid profile, and elevated blood pressure [1]. A key strength of our model is that the relevant hallmarks of the syndrome in animals, such as an increase in retroperitoneal fat pad mass, elevation of serum triglyceride and glucose levels during GTT and ITT, were achieved with high repeatability.

### 3.3. Histopathological and Immunohistochemical Outcomes

In experimental animals, administration of a high-fat high-fructose diet has been found to result in insulin resistance, dyslipidemia, visceral obesity, low-grade inflammation, and oxidative stress [105], which in turn cause distinct histological changes in various organs [106]. In 5 of 10 experiments, we explored and identified morphological changes in the liver, adipose tissue, heart and coronary vessels of rats [60,61,62,65,66,67,77] (Table 5).

Histopathological evaluation of liver samples from metabolic syndrome groups revealed hepatic steatosis, degenerative and necrotic changes in hepatocytes, as well as an inflammatory infiltrate [60,61,66,67,77]. Histopathological changes were associated with biochemical markers for liver damage, such as elevation of ALT, ALP and ALP [67,77]. These results are consistent with the findings described by other authors regarding the effect of metabolic syndrome on the liver [105,107,108]. The mechanisms underlying the described liver changes might be increased *de novo* lipogenesis, hepatic lipid dysregulation, abnormal cytokine production and chronic hepatic inflammation [108]. Additionally, the hyperglycemia brought on by peripheral insulin resistance encourages the liver to produce more fatty acids, which leads to liver steatosis [109]. Cell death is a crucial feature of liver injury. Mitochondrial dysfunction and oxidative stress caused by high blood glucose and lipid overload contribute to the activation of cell apoptosis, as well as damage to cell membranes and proteins in hepatocytes [110,111,112,113,114]. The biomarkers of oxidative stress are increased in many experimental models of metabolic syndrome [51,56,115]. The applied HFHF diet in our experiments led to an enlargement of the visceral adipose tissue and adipocyte size [60,62,66,67,77], as well as to degenerative and inflammatory changes in cardiomyocytes, and to endothelial damage of coronary vessels in the metabolic syndrome groups [60,65,66,67]. The applied diet induces most of the histopathological features of metabolic syndrome that are confirmed in our studies.

#### 3.3.1. Effects of HFHF Diet on Immunohistochemical Markers of Apoptosis

Apoptosis is a tightly regulated process involving extrinsic (receptor-mediated) and intrinsic (mitochondria-dependent) pathways. The intrinsic pathway is governed by Bcl-2 family proteins—pro-apoptotic (e.g., Bax, Bak) and anti-apoptotic (e.g., Bcl-2, Bcl-XL). Upon apoptotic stimuli, Bax/Bak promote mitochondrial membrane permeability and release of pro-apoptotic factors, while Bcl-2 counters these effects to maintain mitochondrial integrity.

Apoptosis plays a key role in inflammation and metabolic dysfunction in adipose tissue [116] and contributes to liver damage in conditions like steatohepatitis and fibrosis [117]. Nutritional status significantly affects mitochondrial apoptosis: caloric restriction improves mitochondrial function, while high-calorie intake and obesity promote apoptosis, especially via Bid-mediated mechanisms [118].

Bax and Bcl-2 expressions were measured in two of our experiments [60,67]. The HFHF diet altered Bax and Bcl-2 expression in the liver and visceral adipose tissue [61,62,67]. Bax levels increased up to 11-fold, and the Bax/Bcl-2 ratio—an indicator of apoptotic susceptibility—was elevated in most tissue samples, suggesting enhanced pro-apoptotic signaling. These findings align with previous studies showing that HFHF diets induce apoptosis to a greater extent than high-fat or high-fructose diets alone [119].

Bcl-2 expression showed inconsistent changes. In one experiment, it was elevated in adipose tissue but remained unchanged in the liver; in another one, the reverse association was observed [60,67]. Most experimental studies report Bcl-2 upregulation in obesity and liver disease, possibly reflecting a compensatory response to metabolic stress or inflammation [120,121]. The regulation of Bcl-2 appears to be context-dependent, influenced by factors such as tissue type, experimental design, disease progression, and activation of inflammatory pathways like NF-κB.

Overall, our data suggest that HFHF diets promote apoptosis primarily through Bax upregulation, while Bcl-2 expression may vary depending on metabolic and inflammatory context, highlighting its complex role in cell survival during obesity.

#### 3.3.2. Effects of HFHF Diet on Immunohistochemical Markers of Inflammation

In two of our experiments, we measured MAC387 liver expression and found that it was increased by the applied HFHF diet [60,67]. MAC387 is a marker for recruited macrophages that infiltrate tissue post-injury, particularly in a CCL2-dependent manner [122]. It distinguishes newly infiltrating macrophages from resident Kupffer cells, as it is predominantly expressed during early monocyte/macrophage differentiation [123]. MAC387-positive cells are typically found in the portal and lobular regions of the liver, especially in areas of interface hepatitis [124]. Increased MAC387 and CD68 expression is a common feature of chronic liver diseases, including MASLD, underlining the key role of macrophages in liver inflammation [125,126].

The HFHF diet elevated MAC387 expression also in the adipose tissue [67]. Obesity is characterized by macrophage infiltration into adipose tissue, which contributes significantly to chronic inflammation [127]. Macrophages and adipose progenitor cells share functional similarities, and in an inflammatory environment, preadipocytes may transdifferentiate into macrophage-like cells [128]. The extent of macrophage infiltration correlates with adipocyte size, body mass index, and C-reactive protein levels [129,130].

The histological changes observed in the liver, adipose tissue, heart, and coronary arteries, along with immunohistochemical evidence of inflammation and altered apoptosis, indicate that our metabolic syndrome model effectively replicates the organ-specific damage commonly associated with the condition in humans. This finding suggests that it could be useful in evaluating different therapeutic strategies related to diet-induced liver or cardiovascular impairment in humans.

### 3.4. Behavioral Outcomes

Locomotor activity was assessed in 9 out of 10 experiments. Across all cases, no significant changes were detected (Table 6). These results suggest that the observed effects in the subsequent behavioral tests are not attributable to changes in the general locomotor activity.

Anxiety-like behavior was evaluated using the OFT, where reductions in the number of entries and the time spent in the central area are indicative of increased anxiety. A significant decrease in the number of center entries was observed in 1 out of 8 experiments (12.5%), while a reduction in the time spent in the center was reported in 3 out of 8 experiments (37.5%). The rats were also tested for anxiety using the SIT in all 10 experiments. In 5 of these, the HFHF animals exhibited anxiety behavior. The decline in social interaction due to high-calorie diets has been attributed to reduced mRNA expression of monoamine oxidase A, catechol-O-methyltransferase, and BDNF in the prefrontal cortex [131]. Alterations in the gut microbiome, which may contribute to the observed behavioral changes, have also been identified [131]. Of the two anxiety tests we utilized, the SIT seems to be more reliable for detecting such behavior.

Depressive-like behavior was evaluated using the FST. In three of the 10 experiments (30%), animals of the HFHF group tested positive. Similar effects in rats exposed to a high-fructose diet were observed during periadolescent development, with associated HPA axis dysregulation and elevated corticosterone levels [132]. Based on our data, the depressive-like behavior in HFHF-fed rats may be linked to increased serum lipid peroxidation [52]. However, despite the clinical evidence of a close inter-relationship between metabolic syndrome and depression, the forced swimming test turns out to be poorly repeatable in our experimental settings.

Cognitive performance was assessed using the NOLT, which evaluates spatial memory. The test was conducted in 6 experiments, and 5 of these (83%) demonstrated deficits in spatial memory. Other authors [133] have also reported reduced discrimination index in NOLT, along with decreased exploratory behavior and lowered hippocampal levels of glutamate and glutamine in rats on an HFHF diet. In another study, impaired long-term memory in both male and female rats subjected to HFHF diet was found, which could be associated with increased oxidative stress biomarkers (malondialdehyde and nitric oxide) [134].

Our experimental studies indicate that spatial memory is significantly affected by the hypercaloric diet. The hippocampus has been identified as one of the brain structures most vulnerable to damage from high-calorie diets [135], and this structure is considered to play a more critical role in spatial memory than in recognition memory [136]. Furthermore, it has been suggested that non-spatial memory may require a longer duration of high-energy diet exposure in rats to show impairment compared to spatial memory [137]. Based on our results, the reduced discrimination index in the NOLT is a finding of low variability, with the spatial memory impairment being repeatable and associated with the diet-induced metabolic syndrome. Thus, the HFHF diet-induced model of metabolic syndrome may represent a useful tool for exploring preventive and therapeutic approaches to obesity-associated cognitive disorders in humans.

### 3.5. Limitations of the Study

Several limitations of our study should be acknowledged. First, it is unexpected that the longest experiment did not produce more pronounced metabolic changes. Indeed, our data suggest that extending the treatment duration from 8 to 15 weeks does not substantially alter the outcomes. We are also unable to explain the absence of an effect in the SIT test in this particular experiment, despite consistent findings in some of the shorter-duration studies.

Another limitation concerns the variability in adipose tissue accumulation. Although an increase in retroperitoneal fat was observed in approximately 80% of the experiments, the remaining 20% still represents a noteworthy level of variability. We cannot provide a rational explanation for this inconsistency, as the species, age, sex of the animals, and treatment duration were comparable across experiments. The variability seen in the other fat pad indices was even greater. We may speculate that differences in genetic predisposition (genetic polymorphism) or specific climatic conditions during the experimental periods might account for these fluctuations, although we currently lack direct data to support these assumptions.

A further limitation is the absence of blood pressure measurements, even though arterial hypertension is a key component of human metabolic syndrome. Finally, in most experiments, HDL-cholesterol levels were not assessed; only total cholesterol was measured. Nevertheless, in the experiments where HDL was evaluated, a decrease consistent with the expected dyslipidemic profile was observed [53,54,58].

## 4. Conclusions

Our experience with the high-fat high-fructose diet-induced model of metabolic syndrome utilized in 10 experimental studies on young adult male Wistar rats shows that it is reliably repeatable, with low variability in most of the somatic, biochemical, histopathological, immunohistochemical, and behavioral data. The HFHF diet applied for 8–15 weeks does not increase the final body weight. However, it leads to the development of visceral obesity as a good estimate of metabolic syndrome. Food consumption decreases but caloric intake increases. Among the repeatable biomarkers, the increased plasma triglyceride levels, as well as the elevated glucose levels during GTT and ITT, appear to be reliable findings. Elevation of fasting blood glucose and total cholesterol levels are less repeatable. The histopathological findings of hepatic steatosis, degenerative and necrotic changes, and inflammatory infiltrates are supported by increased biomarkers for liver damage and positive immunohistochemical tests for apoptosis and inflammation in separate experiments. The behavioral changes resembling anxiety and depression appear to be insufficiently repeatable. The impairment of spatial memory is a relatively consistent cognitive feature of the HFHF diet-induced metabolic syndrome. We may conclude that the presented high-fat, high-fructose diet-induced model of metabolic syndrome could serve as a valuable tool for evaluating therapeutic strategies—such as dietary interventions and pharmacological treatments—aimed at improving human health.

## Figures and Tables

**Table 1:** Design of the experimental studies with Wistar rats subjected to a high-fat high-fructose (HFHF) diet conducted by our research team.

Experiment №	Duration in Weeks	Number and Description of Experimental Groups	Number of Animals per Group	Brief Description of the Aim of the Experiment	References
1	8	3 (a control group, an HFHF group, and a group fed another hypercaloric diet)	12	To verify a model of metabolic syndrome	[51,52,53]
2	10	4 (a control group, an HFHF group, and 2 groups treated with vitamin K2)	10	To study the effects of vitamin K2 in a model of metabolic syndrome	[51,52,53,54,55]
3	10	2 (a control and an HFHF group)	10	To study the pathophysiological role of osteocalcin in a model of metabolic syndrome	[53,56]
4	11	4 (a control group, an HFHF group, and 2 groups treated with warfarin)	12	To study the effects of warfarin on osteocalcin and metabolic syndrome	[53,57,58]
5	15	4 (a control group, an HFHF group, and 2 groups treated with alendronate)	12	To study the effects of alendronate on osteocalcin and metabolic syndrome	[53,59]
6	10	5 (a control group, an HFHF group, and 3 groups treated with 3 doses of *Aronia melanocarpa* fruit juice)	10	To study the effects of *Aronia melanocarpa* on metabolic syndrome	[60,61,62,63,64]
7	10	5 (a control group, an HFHF group, and 3 groups treated with 3 doses of *Chaenomeles maulei* fruit juice)	10	To study the effects of *Chaenomeles maulei* on metabolic syndrome	[65,66]
8	10	5 (a control group, an HFHF group, and 3 groups treated with 3 doses of anethole)	10	To study the effects of anethole on obesity	[67,68,69,70,71,72,73,74]
9	12	5 (a control group, an HFHF group, and 3 groups treated with 3 doses of *Kochia scoparia* seed infusion)	10	To study the effects of *Kochia scoparia* on metabolic syndrome	[75,76]
10	9	6 (a control group, an HFHF group, and 4 groups treated with different *Aronia melanocarpa*-based juices)	10	To study the effects of different *Aronia melanocarpa*-based juices on metabolic syndrome	[77]

**Table 2:** Changes (% of change compared to control) in food, liquids and calorie consumption, and body weight in Wistar rats subjected to a high-fat high-fructose diet. gray, not measured; red, significant change of the parameter; green, no significant change of the parameter; *n*, number of animals per group.

		% of Change		
Experiment №	Reduced Food Consumption	Increased Consumption of Liquids	Increased Calorie Intake	Change of Final Body Weight
1 (*n* = 12)	38.43	37.08	15.42	3.44
2 (*n* = 10)				0.54
3 (*n* = 10)				5.72
4 (*n* = 12)	36.74	29.42	19.64	6.04
5 (*n* = 12)	38.48	42.56	19.00	3.42
6 (*n* = 10)				1.79
7 (*n* = 10)	32.58	47.12	28.79	6.89
8 (*n* = 10)	31.93	32.25	32.76	1.25
9 (*n* = 10)	36.72	45.05	24.24	3.70
10 (*n* = 10)	41.36	87.29	21.17	4.33
Average	36.7	45.1	22.6	3.75

**Table 3:** Changes (% of change compared to control) in visceral obesity of Wistar rats subjected to high-fat high-fructose diet. gray, not measured; red, significant increase of the fat pad index; green, no significant change of the fat pad index; *n*, number of animals per group.

			% of Change		
Experiment №	Retroperitoneal Fat Pad Index	Mesenteric Fat Pad Index	Paranephric Fat Pad Index	Perigonadal Fat Pad Index	Total Visceral Fat Index
1 (*n* = 12)	126.93				
2 (*n* = 10)	36.54				
3 (*n* = 10)	47.48				
4 (*n* = 12)	36.71				
5 (*n* = 12)	50.54				
6 (*n* = 10)	49.25	7.81	23.53	12.36	29.46
7 (*n* = 10)	93.07	68.1	68.56	38.03	66.19
8 (*n* = 10)	63.41	69.32	26.71	19.03	45.61
9 (*n* = 10)	25.71	21.33	16.66	3.26	13.44
10 (*n* = 10)	33.33	21.57	23.81	0.99	11.15
Average	57.15	37.63	31.85	14.73	33.17

**Table 4:** Changes (% of change compared to control) in plasma biochemical markers in Wistar rats subjected to a high-fat high-fructose diet.

			% of Change		
Experiment №	Triglycerides	Total Cholesterol	Fasting Blood Glucose	GTT (Blood Glucose Level After Glucose Administration)	ITT (Blood Glucose Level After Insulin Administration)
1 (*n* = 12)	215.57	36.20	7.13		20.81
2 (*n* = 10)	18.65	3.76	11.85		
3 (*n* = 10)	20.97	6.56	8.54		16.27
4 (*n* = 12)	8.49	5.80	10.68	30.3	
5 (*n* = 12)			6.59	26.88	7.87
6 (*n* = 10)	51.92			34.99	
7 (*n* = 10)	82.62	13	2.28	33	
8 (*n* = 10)	19.7	11.57	2.96	24.18	
9 (*n* = 10)	46.75	27.35		14.86	
10 (*n* = 10)	46.91	26.14	0.59	21.01	
Average	59.19	16.51	6.45	26.58	14.91

GTT, glucose tolerance test; ITT, insulin tolerance test; gray, not measured; red, significant elevation/impairment of the parameter; green, no significant change of the parameter; *n*, number of animals per group.

**Table 5:** Histopathological changes in liver, adipose tissue, heart and coronary blood vessels of Wistar rats subjected to high-fat high-fructose diet. gray, not evaluated; red, presence of histopathological changes.

Experiment №	Liver (Microvesicular Steatosis, Ballooning Degeneration, Non-Specific Inflammatory Granulomas)	Adipose Tissue (Adipocyte Hypertrophy)	Heart (Cardiomyocyte Degeneration, Inflammation and/or Necrosis)	Coronaries (Endothelial Damage and/or Activation)
1				
2				
3				
4				
5				
6				
7				
8				
9				
10				

**Table 6:** Behavioral changes (% of change compared to control) in Wistar rats subjected to a high-fat high-fructose diet. OFT, open field test; SIT, social interaction test; FST, forced swim test; NOLT, novel object location test; gray, not measured; red, significant change of the parameter; green, no significant change of the parameter; *n*, number of animals per group.

			% of Change			
Experiment №	OFT (Locomotion)	OFT (Decreased Time in Center)	OFT (Decreased Number of Entries in the Center)	SIT (Decreased Time of Social Interaction)	FST (Increased Immobility Time)	NOLT (Decreased Discrimination Index)
1 (*n* = 12)	6.03	36.54	70.80	12.12	20.97	
2 (*n* = 10)				43.74	20.40	
3 (*n* = 10)	38.12			43.95	9.49	
4 (*n* = 12)	10.96	15.14	17.50	36.34	11.87	31.09
5 (*n* = 12)	7.14	6.55	0	31.17	9.05	19.29
6 (*n* = 10)	34.43	44.69	140	28.41	24.82	32.31
7 (*n* = 10)	22.91	21.17	21.43	20.37	6.28	22.27
8 (*n* = 10)	38.59	40.03	35.71	30.40	7.27	32
9 (*n* = 10)	12.65	52.65	47.36	32.88	7.84	19.7
10 (*n* = 10)	43.21	42.22	57.26	2.63	35.93	
Average	22.80	31.47	45.36	28.11	15.31	26.05

## Data Availability

The original data presented in the study are openly available in the original articles that are cited in this study [51,52,53,54,55,56,57,58,59,60,61,62,63,64,65,66,67,68,69,70,71,72,73,74,75,76,77].

## Abbreviations

The following abbreviations are used in this manuscript:

ALT	Alanine aminotransferase
ALP	Alkaline phosphatase
AST	Aspartate aminotransferase
BDNF	Brain-derived neurotrophic factor
BWG	Body weight gain
CI	Caloric intake
FST	Forced swim test
GTT	Glucose tolerance test
HFHF	High-fat high-fructose
HPA	Hypothalamic–pituitary–adrenal
ITT	Insulin tolerance test
MASLD	Metabolic Dysfunction-Associated Steatotic Liver Disease
nm	Not measured
NOLT	Novel object location test
OFT	Open field test
SIT	Social interaction test
TBARS	Thiobarbituric acid-reacting substances
TG	Triglycerides
